# Validation of Molecular Typing for Endometrial Screening Test That Predicts Benign and Malignant Lesions

**DOI:** 10.3389/fonc.2019.00561

**Published:** 2019-07-09

**Authors:** Xiaoqian Tuo, Lanbo Zhao, Qi Wang, Lu Han, Yiran Wang, Sijia Ma, Xue Feng, Qing Li, Chao Sun, Qing Wang, Guizhi Shi, Huilian Hou, Guanjun Zhang, Qiling Li

**Affiliations:** ^1^Department of Gynecology and Obstetrics, First Affiliated Hospital, Xi'an Jiaotong University, Xi'an, China; ^2^Guipei 77, Health Science Center, Xi'an Jiaotong University, Xi'an, China; ^3^Aviation General Hospital of Beijing, Medical University and Beijing Institute of Translational Medicine, University of Chinese Academy of Sciences, Beijing, China; ^4^Department of Pathology, First Affiliated Hospital, Xi'an Jiaotong University, Xi'an, China

**Keywords:** immunocytochemistry, Ki-67, p53, CA125, probit model

## Abstract

The aim of this study is to examine the immunocytochemical expression of p53, Ki-67, and CA125 in endometrial brush samples for endometrial cancer. Forty-four patients were recruited with liquid-based cytology preparations during a 5-month period. Both the histological and cytological samples were assessed by histology based on hematoxylin and eosin (H&E), and the expression of p53, CA125, and Ki-67 in endometrial cells was examined by immunocytochemistry. The percentage and intensity of endometrial cells were scored on a scale of 0–3. The final score was calculated by the addition of all partial scores, and then Probit model was used to predict the possibility for malignant lesions. The mean immunoreactivity score of the three immunocytochemical biomarkers (p53, CA125, and Ki-67) in the positive group (including atypical hyperplastic cells and malignant cells) was significantly higher than in the negative group (benign cells and non-atypical hyperplastic cells). The possibility value of the positive group was also significantly higher than the negative group (*P* < 0.05). The cutoff value of the possibility value was 0.754, the sensitivity and specificity of which were 86.4 and 95.5%. The assessment of p53, CA125, and Ki-67 combined with the prediction model is valuable for the detection of endometrial cancer and atypical hyperplasia in endometrial cytology.

## Introduction

Cancer of the uterine corpus is the most common female pelvic cancer in western countries, approximately accounting for 4.4% of cancers in women and approximately 382,069 estimated new endometrial cancer cases worldwide in 2018 (1). It predominantly occurs in women after menopause, and the estimated age-standardized incidence rates (ASRs) vary from 1 to 30 cases per 100,000 women across all nations (2, 3). The 5-year survival rate of endometrial carcinoma decreases with the development of endometrial carcinoma stages, and previous study shows that 80% of the patients diagnosed with endometrial cancer are stage I, with a >95% 5-year survival rate (4). Therefore, a simple, accurate and cost-effective screening method to detect endometrial atypical hyperplasia and the early stages of endometrial cancer is an urgent need in clinical practice (5). At present, the gold standard for the diagnosis of EC is to obtain a tissue specimen for pathological results via dilatation and curettage (D&C) under hysteroscopy (6). However, D&C is a fairly invasive method with poor compliance, limitations of accuracy and long-term monitoring difficulties (7, 8).

Over the years, endometrial sampling brush devices have been developed for use in the endometrial cytology test (ECT) (9–11), such as Li Brush, SAP-1 and Tao Brush (10, 12). Endometrial liquid-based cytology has been widely used for a long time (13–17). ECT presents the advantages of high satisfaction, reduced pain and bleeding, convenient operation, economic efficiency, and high safety. However, limitations exist in ECT, such as that early-staged and well-differentiated endometrioid adenocarcinoma cases present the possibility of being misdiagnosed (18). Furthermore, there is not yet a unified standardization system and the diagnosis is largely associated with cell morphology, whereas transformation of cells occurs due to centrifugation during cytopreparation that complicates the diagnostic accuracy. Recent studies of immunocytochemical assessment imply that this method could increase the diagnostic accuracy and might be a potential and promising method for the early detection of EC and precancerous lesions (19, 20).

The aim of this study was to investigate the validity of certain immunocytochemical biomarkers as a screening method for menopausal women with high-risk factors. We assessed the immunocytochemical expression within endometrial samples by using p53, CA125, and Ki-67 antibodies in liquid-based cytology preparations. Moreover, we modeled a formula to predict the possibility for malignant lesions by using the combination of these markers.

## Materials and Methods

### Patients

During a 5-month period (08/2017 to 01/2018), 44 patients were enrolled for endometrial cytology testing at the Department of Obstetrics and Gynecology in the First Affiliated Hospital of Xi'an Jiaotong University. This experimental protocol was approved by the Ethics Committee of the First Affiliated Hospital of Xi'an Jiaotong University, with written consent obtained from all patients. The study comprised the patients who underwent total hysterectomies or D&Cs. Furthermore, the patients' final diagnoses were confirmed by postoperative histopathologic examinations. Patients with consistent diagnosis of cytology and histology based on hematoxylin and eosin (H&E) staining were enrolled in this study. The histological diagnostic types, according to the International Society of Gynecological Pathology Classification, included endometrial carcinoma, hyperplasia with atypia, hyperplasia without atypia, atrophic endometrium, proliferative endometrium, and secretory endometrium (21). The cytological diagnosis was made according to the criteria formulated by Fox, ranging from benign cells (including atrophic, proliferative and secretory endometrium), non-atypical hyperplastic cells, and atypical hyperplastic cells to malignant cells (22). In addition, atypical hyperplastic cells and malignant cells were diagnosed as positive results, while others were addressed as negative.

### Cytologic and Histological Preparations

All endometrial cytologic samples were collected from patients using the Li Brush (20152660054, Xi'an Meijiajia Medical Technology Co. Ltd., China). The brush was designed to be similar in shape to the uterine cavity and was supposed to be capable of collecting more endometrial cells and providing more accurate diagnosis of endometrial lesions (10). The brush was pushed into the uterus until it reached the fundus, and the sheath was retracted to expose its broom head to the uterine cavity. The brush was rotated approximately 5 to 10 circles clockwise, and the sheath was placed over the broom head to protect the collected endometrial cells. Then, the brush was removed from the uterus and the brush head was immediately immersed into the endometrial preservation solution in the vial. The vial was labeled after the brush was vibrated more than 5 times and discarded.

The vials were vortex mixed to resuspend sedimented cells. The suspension was placed into a 15-mL centrifuge vial, and after centrifugation at 2,400 rpm for 3 min, the supernatant was discarded and the pellet was placed into a 50-mL centrifuge tube. The pellet was then resuspended using the BD CytoRich™ Red Preservative (491336, BD Biosciences, USA) to lyse red blood cells and solubilize proteins while preserving the diagnostically relevant cells. Centrifugation was performed again at 600 rpm for 10 min. The supernatant was discarded and buffer solution (490518, BD Biosciences, USA) was subsequently added. Centrifugation was performed one additional time at 600 rpm for 5 min. The supernatant was discarded and the remnant was resuspended with buffer solution. We dripped 1-2 sample drops onto the PreCoat Slide (491238, BD Biosciences, USA), and the slides were then left to cure naturally for 30 min. Finally, the slides were prepared for HE staining and immunocytochemical staining after being fixed via 95% ethanol.

The histological samples were collected from the patients during surgery and D&C. The removed uteri or scratched tissues were fixed in 10% buffered formalin and routinely dehydrated, hyalinized, wax-impregnated, embedded, and sliced. Then, slices were investigated histologically using hematoxylin and eosin staining.

### Hematoxylin and Eosin (H&E) Staining

The cytological slides were immersed in H_2_O with agitation for 3–5 min to hydrate the cells and were then dipped into a Coplin jar containing Mayer's hematoxylin for 2 min. The slides were then rinsed in H_2_O for 1 min. Afterwards, slides were stained with 1% eosin Y solution for 1 min with agitation. The dehydration step was performed using four changes of 95% alcohol and two changes of 100% alcohol for 30 s each. Then, the alcohol was extracted with two changes of xylene for 30 s each. In the end, slides were sealed with neutral resin.

The histological slices were sequentially immersed in xylene, anhydrous ethanol, 95% alcohol and 80% alcohol to dewax and then washed 3-5 min with water. The following steps were consistent with the cytological slides.

### Immunocytochemistry (ICC) for p53, CA125, Ki-67

The ICC staining was performed using an autostainer (XT System Benchmark, Roche Diagnostics, Basel, Switzerland). The primary antibodies used in this report were a mouse monoclonal antibody against p53 (Kit-0010; clone D0-7; Maxim Biotechnologies, Fuzhou, China), a mouse monoclonal antibody against CA125 (ZM-0019; Zsbio; China), and a mouse monoclonal antibody against Ki-67 (Kit-0005; Maxim Biotechnologies, China). Next, the ultraView Universal DAB Detection Kit (760-500; Roche Diagnostics, Switzerland) was used to detect primary antibodies indirectly. All steps were performed according to the standard procedures provided by the kits.

The staining results were evaluated by two experienced pathologists using conventional optical microscopy. The distribution and intensity of staining cells in immunocytochemical stained slides were observed under light microscope. The staining cytoplasm and/or nucleus exhibited brown-yellow particles, and the staining intensity was significantly higher than that of the background. For the evaluation of p53 and Ki-67 immunoreactivity, the intensity of nuclear staining and the nuclear labeling index (N-LI) were taken into consideration. At least 500 cells in randomly selected fields were counted. The intensity of nuclear staining was scored as negative (-) (0; Figure 2D), weak (+) (1; Figure 2C), moderate (++) (2; Figures 2B,F–H), or strong (+++) (3; Figures 2A,E). The N-LI was scored as <10% (0), from 10 to 39% (1), from 40 to 69% (2), or >70% (3). For the evaluation of CA125 immunoreactivity, both the percentage of cytoplasm staining and the intensity of staining were estimated. Intensity of immunostaining was scored as negative (–) (0; Figure 2L), weak (+) (1; Figure 2K), moderate (++) (2; Figure 2J), or strong (+++) (3; Figure 2I), according to a four-tiered scale based on the predominant staining intensity in endometrial cells. The percentage of staining cells was scored as <10% (0), from 10 to 39% (1), from 40 to 69% (2), or >70% (3).The final immunoreactivity score was calculated by the addition of both partial scores (23).

### Prediction Model

The response variable that is classified into positive or negative groups is a binary or dichotomous variable. Since the binary regression models are developed to predict a binary dependent variable as a function of predictor variables, they are applicable techniques to predict the possibility of being diagnosed as positive or negative. We choose to employ a random parameter binary Probit model to allow for the potential correlation with respect to unobserved factors. In a random parameter model, some or all of the parameters are assumed to be random and will vary across observations (24). The random parameter binary Probit model is later estimated using Eviews (25–27). When the distribution function adopts the standard normal distribution, the Probit model with the explanatory variables of p53 immunoreactivity score (X1), CA125 immunoreactivity score (X2), Ki-67 immunoreactivity score (X3) and the dependent variable is obtained: Y = 1−@CNORM[−(−8.04545212047 + 0.732971762404^*^X1 + 0.468241629323^*^X2 + 0.652156496038^*^X3)].

We then introduced the Gaussian, or “normal” distribution:

$$\begin{array}{l} f\left(x\right)=\frac{1}{\sqrt{2\pi }}e^{\left(-\frac{x^{2}}{2}\right)} \end{array}$$

where f(x) is the possibility value, and “x” is the Y in the former formula. Finally, we obtained the possibility value of each sample and then used the ROC curve for assessment.

### Statistics

The final score of intensity of the ICC staining was calculated by the addition of both scores. Statistical Package for the Social Sciences version 18.0 (SPSS, Chicago, IL) was used in this study. Eviews were used for statistical analysis, such as the *t*-test and receiver operating characteristic curve (ROC curve). A *P*-value below 0.05 was considered to be statistically significant.

## Results

### Patients

Forty-four patients in total (mean age: 51.4 years, range 24–77 years) were enrolled in this study. Among these, there were 20 endometrial cancer and 2 atypical hyperplasia samples in the positive group (P). Their median age was 55.4 years (range: 24–72 years). Correspondingly, 22 patients with benign cells and non-atypical hyperplastic cells constituted the negative group (N), whose median age was 47.4 years (range: 30–77 years). Those included 15 proliferative endometrium, 3 secretory endometrium and 4 atrophic endometrium samples.

### Hematoxylin and Eosin (H&E) Staining

**Figure 1:**
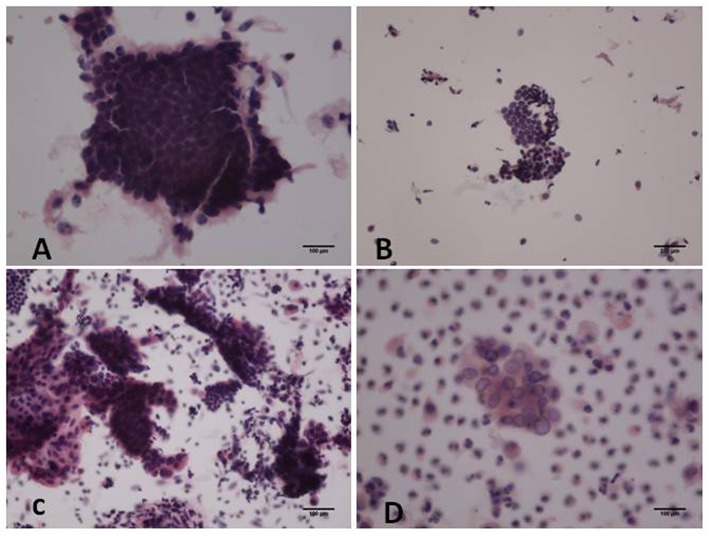
Hematoxylin and eosin (H&E) staining of cytological samples. **(A)** Proliferative endometrium; **(B)** Atrophic endometrium; **(C)** Atypical hyperplasia with localized carcinogenesis; **(D)** Highly differentiated endometrioid carcinoma [**(A,C-D)**: original magnification X200; **(B)**: original magnification X100].

In the proliferative phase, the size and shape of the nucleus were uniform and the nucleus was deeply stained. More cells and cell communities were observed. Furthermore, cell nuclei were crowded and arranged in a polar orientation (Figure 1A). Figure 1B shows fewer cell colonies, along with small and deeply stained nuclei in the atrophic phase. Irregularly shaped cell communities with non-uniform nuclei and irregular shapes were observed in atypical hyperplasia with localized carcinogenesis (Figure 1C). Highly differentiated endometrioid adenocarcinoma in Figure 1D was diagnosed due to a community distribution of cancer cells presenting irregular nuclei and nucleoli.

**Figure 2:**
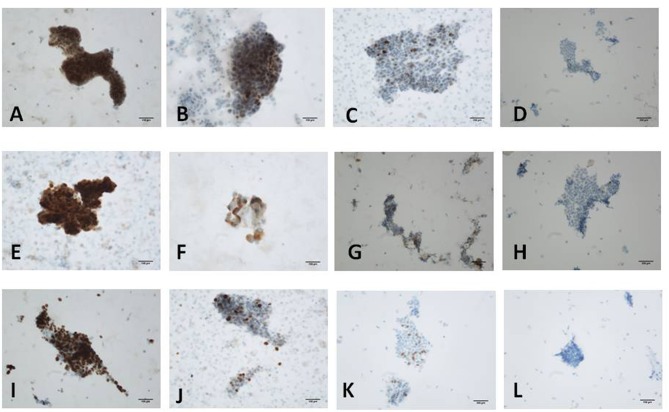
Immunostaining for p53, Ki-67 and CA125 protein. **(A)** Scored 6 of p53 in moderately differentiated endometrioid carcinoma (+++, 90%); **(B)** Scored 4 of p53 in localized serous carcinoma (++, 40%); **(C)** Scored 2 of p53 in proliferative endometrium (+, 10%); **(D)** Scored 0 of p53 in atrophic endometrium (–). **(E)** Scored 6 of Ki-67 in moderately differentiated endometrioid carcinoma (+++, 70%); **(F)** Scored 4 of Ki-67 in atypical hyperplasia with carcinogenesis (+++, 30%); **(G)** Scored 3 of Ki-67 in proliferative endometrium (++, 30%); **(H)** Scored 2 of Ki-67 in atrophic endometrium (++, 1%). **(I)** Scored 6 of CA125 in atypical hyperplasia with carcinogenesis (+++, 70%); **(J)** Scored 5 of CA125 in endometrial serous carcinoma (++, 70%); **(K)** Scored 2 of CA125 in atrophic endometrium (+, 20%); **(L)** Scored 0 of CA125 in proliferative endometrium (−) [**(A-C,E-G,I,J,L)**: original magnification X200; **(D,H,K)**: original magnification X100].

### ICC Staining for p53, CA125, Ki-67

Endometrial cell types on slides were confirmed according to nuclear size, nuclear/cytoplasmic ratio, and nuclear membrane after H&E staining combined with the histopathologic reports. Immunocytochemistry showed that p53 and Ki-67 were primarily expressed in the nucleus (Figures 2A–H) and that CA125 was mainly expressed in the cytoplasm (Figures 2I–L). The comparison of p53, CA125, and Ki-67 immunoreactivity scores is shown in Table 1. For p53 protein immunoreactivity, final immunoreactivity scores of 0, 1, 2, 3, 4, 5, and 6 accounted for 0, 0, 5, 9, 14, 14, 14, and 59% in the positive group, respectively, while in the negative group, p53 final immunoreactivity scores of 0, 1, 2, 3, 4, 5, and 6 accounted for 5, 18, 50, 9, 14, 5, and 0%, respectively. Additionally, mean p53 protein immunoreactivity scores of the P Group (5.14 ± 1.25) exhibited significantly higher values (*P* < 0.05, Table 1) in comparison with the N Group (2.23 ± 1.19).

**Table 1:** Results of *t* test showing the immunoreactivity scores' correlations between positive group and negative group.

	**Group**	**Mean**	**Std. deviation**	**Std. error mean**
p53	P	5.1364	1.24577	0.26560
	N	2.2272	1.19251	0.25424
Significance				*P*_1_^*^ <0.05
CA125	P	5.2727	1.12045	0.23888
	N	3.6364	1.91598	0.40849
Significance				*P*_2_^*^ = 0.001
Ki67	P	5.5909	0.66613	0.14202
	N	4.2273	1.44525	0.30813
Significance				*P*_3_^*^ <0.05

*P: Positive Group (including atypical hyperplastic cells and malignant cells)*.

*N: Negative Group (including non-atypical hyperplastic cells and benign cells)*.

P_1_^*^: P value for t test of p53 between the Positive Group and Negative Group.

P_2_^*^: P value for t test of CA125 between the Positive Group and Negative Group.

P_3_^*^: P value for t test of Ki67 between the Positive Group and Negative Group.

As for CA125 immunoreactivity, final scores of 0, 1, 2, 3, 4, 5, and 6 accounted for 0, 0, 0, 14, 9, 14, and 64% in the P Group, respectively, while within the N Group, the CA125 immunoreactivity scores of 0, 1, 2, 3, 4, 5, and 6 accounted for 9, 0, 23, 18, 9, 18, and 23%, respectively. In addition, mean CA125 immunoreactivity scores of the P Group (5.27 ± 1.12) were of significantly higher values (*P* < 0.05, Table 1) in comparison with the N Group (3.64 ± 1.92).

As for Ki-67 immunoreactivity, the final immunoreactivity scores of the Positive Group were distributed from 4 to 6 and accounted for 9, 23, and 68%, respectively, while in the Negative Group the final immunoreactivity scores of 0, 1, 2, 3, 4, 5, and 6 accounted for 0, 5, 9, 18, 14, 36, and 18%, respectively. The P group (5.59 ± 0.67) exhibits significantly higher values (*P* < 0.05, Table 1) in comparison with the N group (4.23 ± 1.45). All of these findings were statistically significant.

Finally, through the comprehensive evaluation of the above p53, CA125, and Ki-67 immunoreactivity scores together with the normal distribution function, the possibility value of a sample was acquired. Additionally, when the possibility value of a sample was close to 1, this indicated that the sample was more likely to belong to the positive group, such as atypical hyperplastic cells and malignant cells. In contrast, when the possibility value of a sample was close to 0, it indicated that the sample was likely to belong to the negative group such as benign cells and non-atypical hyperplastic cells. The possibility values of the positive group were 0.87 ± 0.24, in comparison with 0.17 ± 0.28 in the negative group, and the positive group demonstrated statistically significant differences compared with the negative group (*P* < 0.05, Table 2).

**Table 2:** Results of possibility values between Positive Group and Negative Group.

	**Group**	**Mean**	**Std. deviation**	**Std. error Mean**	**Significance**
Possibility value	P	0.8711	0.2354	0.05018	<0.05
	N	0.1672	0.2800	0.05970	

*P: Positive Group (including atypical hyperplastic cells and malignant cells)*.

*N: Negative Group (including non-atypical hyperplastic cells and benign cells)*.

**Figure 3:**
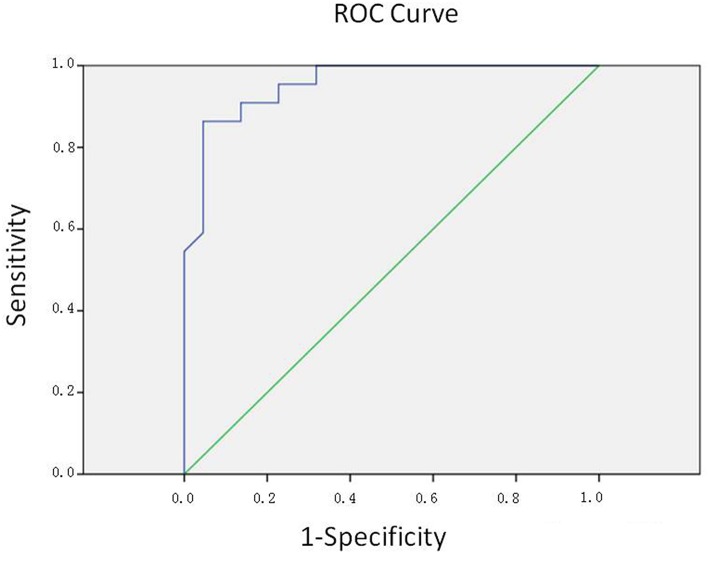
ROC curve. The area under the ROC curve (AUC) is 0.956, indicating a highly accurate test.

Since we tended to use the model as a screening method, the efficiency was evaluated by the area under the ROC curve (AUC) of the receiver operating characteristics (ROC) curve. The area under the ROC curve (AUC) is 0.956, which indicated a highly accurate test according to an arbitrary guideline [based on a suggestion by Swets (28, 29)] (Figure 3). The cut-off value was decided according to the Youden index, which was calculated via standard specificity and sensitivity from the ROC curve. Accordingly, we selected the cut-off value of 0.754, when the sensitivity was 86.4%, and the specificity was 95.5%.

## Discussion

Evidence shows that cytological examination with respect to the detection of endometrial lesions is much simpler to perform than histopathological examination. Although the application of endometrial cytology is rising in clinical and scientific research, there is not yet a unified standardization system, diagnosis is largely associated with the cell morphology, and cytological samples are rather complicated to interpret (30–32). Previous research indicates that the evaluation of immunocytochemistry, in addition to cytomorphologic features, appears to be valuable for the detection of endometrial carcinoma in endometrial cytology (16, 19, 33). Therefore, we comprehensively assess the expression of p53, CA125, and Ki-67 in variable endometrium samples and propose a model that might be applied to the diagnosis of benign and malignant cytology.

Among these, p53 works as a tumor suppressor gene that occurs most frequently in human neoplasia. It has been illustrated that mutation of p53 plays a vital role in tumor progression in malignant lesions, including endometrial carcinoma. The mutated form of p53 produces a non-functional protein that allows for immunocytochemical detection (16, 34). In this study, the analysis of the p53 expression indicates that atypical hyperplastic cells and malignant cells had higher immunoreactivity scores. This result is consistent with previous studies in which the overexpression of p53 is found to be associated with high histologic grade and is significantly higher in grade 3 endometrial cancer (35). Previous research also indicated that p53 was not expressed in normal endometrium and that overexpression of p53 was significantly higher in grade 3 tumors (36). Furthermore, overexpression of p53 is related to poor prognostic indicators (2, 37–39). Consequently, the expression of nuclear p53 protein may be of value in the cytological diagnosis of EC. However, weak expression of p53 is also observed in several hyperplasia endometrium samples, which might be a consequence of DNA damage (40).

As for CA125, prior studies regarding immunohistochemical staining demonstrate that the majority of endometrioid endometrial carcinoma tissues contain CA125 (41). Previous studies indicate that CA125 is a secretory product of normal endometrium and is presented in normal endometrial samples, especially during the secretory phase (42–44). Evidence of immunohistochemistry indicates that the staining pattern and intensity in hyperplasia with atypia can help differentiate this condition from well-differentiated endometrioid carcinoma and that CA125 could be a marker of malignant cell transformation (45, 46). However, there exists little research about immunocytochemical expression with respect to CA125 in malignant and benign endometrial samples. The results in this study show that the immunoreactivity scores of P Group are significantly higher than N Group (*P* < 0.05). However, in the N Group, the immunoreactivity scores that scatter equally from 0 to 6 lead to confusion with respect to differentiation of endometrial samples. We therefore consider that the immunoreactivity score of CA125 alone may not be suitable to differentiate malignant lesions. Combined application of other biomarkers may provide more accurate results.

Ki-67 is a cell proliferation marker expressed in all phases of the cell cycle, except G0, and has been reported to reflect the growth fraction in neoplastic lesions (47). It has been indicated as an important marker in the field of evaluation of endometrial carcinoma in previous findings (48, 49). Ki-67 is proven to be a diagnostic stain of high importance for secretory and atrophic endometrium (19). However, Ki-67 alone seems to be an unreliable marker for proliferative endometrium, as it presents similar immunoreactivity scores with malignant endometrial lesions. Consequently, Ki-67 alone may also not be a promising marker for differentiating malignant endometrial lesions from proliferative endometrium.

For this reason, we attempted to assess the validation of these biomarkers used for the endometrial screening test that differentiate benign, malignant, and precancerous lesions. We first used the immunocytochemical data in this study to construct a random parameter binary Probit model to predict the possibility value of a sample belonging to the positive or negative group. The cut-off value was 0.754 when the sensitivity was 86.4% and the specificity of 95.5% was chosen. However, additional data are required to be added into this model in order to obtain a more ideal model and to assess the model for further verification.

We evaluated the expression of p53, CA125, and Ki-67 in variable endometrial samples by using the endometrial sampling device and liquid-based cytology. Furthermore, we modeled to predict the possibility of sample malignancies. The findings showed that the cut-off value of 0.754 was apparently useful for a more accurate diagnosis, since the sensitivity was 86.4%, and the specificity was 95.5%. Meanwhile, the endometrial sampling device and liquid based cytology were more practical and harmless, making them promising alternatives to other methods. The assessment of p53, CA125, and Ki-67 combined with the prediction model might be valuable for the detection of endometrial cancer and atypical hyperplasia in endometrial cytology.

## Data Availability

The raw data supporting the conclusions of this manuscript will be made available by the authors, without undue reservation, to any qualified researcher.

## Ethics Statement

This experimental protocol was approved by the Ethics Committee of the First Affiliated Hospital of Xi'an Jiaotong University, with written consent obtained from all patients.

## Author Contributions

QilL was the PI of the study and participated in design, analysis. XT and LZ performed the bench work and drafted the manuscript. QiW, LH, YW, SM, XF, and QinL helped to collect the samples. CS and QinW helped to perform the statistic analyses. GS, HH, and GZ performed the pathologic reports.

### Conflict of Interest Statement

The authors declare that the research was conducted in the absence of any commercial or financial relationships that could be construed as a potential conflict of interest.
